# A pilot study of a novel, incentivised mHealth technology to monitor the vaccine supply chain in rural Zambia

**DOI:** 10.11604/pamj.2019.33.50.16318

**Published:** 2019-05-22

**Authors:** Camillo Lamanna, Lauren Byrne

**Affiliations:** 1 Division of Emergency Medicine, University of Cape Town, Cape Town, South Africa; 2 Department of Emergency Medicine, University of Sydney, Sydney, Australia

**Keywords:** mHealth, supply chain management, vaccine logistics

## Abstract

**Introduction:**

The World Health Organization estimates that up to half of vaccines are wasted, however only a minority of mHealth programs in Africa have been directed at vaccine supply chain optimisation. We piloted a novel mHealth solution dependent only on short message services (SMS) technology that allowed workers in rural health centres in Zambia to report vaccine stock levels directly to an online platform. Small airtime incentives were offered to encourage users to engage with the system, as well as weekly reminder messages asking for stock updates.

**Methods:**

The primary outcome measured was the percentage-of-doses-tracked, calculated over the study period. Each vaccine box was randomly allocated to offer either a standard or double airtime incentive and either weekly or daily reminders, in a 2 x 2 design; ANOVA was used to calculate if any of these factors affected time-to-reply.

**Results:**

Over the study period, the total percentage-of-doses-tracked was 39.9%. Within the subset of users who sent at least one message to the platform, the percentage-of-doses-tracked was 93.8%. There was no significant difference in average time-to-reply between the standard airtime incentive and double airtime incentive groups, nor was there a significant difference between the standard reminder and daily follow-up reminder groups.

**Conclusion:**

This pilot study found that in an active subgroup of health workers, an incentivised mHealth solution was able to collect tracking data for 93.8% of doses. More research is needed to identify methods to encourage healthcare workers to engage in timely stock reporting practices.

## Introduction

In the past decade, mobile phone ownership and mobile network infrastructure have rapidly expanded across sub-Saharan Africa. In tandem with this, there have been numerous initiatives to use new mobile health (mHealth) technologies to improve healthcare in the region. These have ranged from reminders [1], through to delivery of care [2], diagnostic pathways [3] and surveillance programs [4]. While the World Health Organization estimates that up to 50% of vaccines are wasted [5], only a minority of mHealth programs in Africa have been directed at vaccine supply chain optimisation. However, there have been successful pilots using mHealth technology to monitor supply chains for other medications [6, 7]. Indeed, with the introduction of newer, more expensive vaccines, the need for improved supply chains and logistics systems is greater than ever [8]. In Zambia, prior to 2015, vaccine stock levels were recorded manually using paper registries. There was no timely co-ordination of these individual registries at national level; information was often out-of-date by the time it had been received at the central warehouse in Lusaka [9]. In response to this situation, the Zambian Ministry of Health launched in 2015 a pilot using mHealth technology to enable real-time reporting of vaccine stocks. Over the course of 2016-7, the pilot was expanded and successfully rolled out across all provincial and district health facilities [10]. However, the project faced two main challenges.

Firstly, the mHealth intervention required Java- or smart-phones which could connect to mobile data networks. While the prevalence of mobile phones in Zambia is high and always increasing, rural smartphone penetration is low and access to mobile data in rural areas is highly variable [11]. This is a challenge for all mHealth supply chain management systems to operate effectively over the “last mile.” Secondly, the reporting rate over the period 2016-7 was between 50-70%. Low uptake was felt to be multifactorial in nature, with proposed causes including a lack of sufficient training and understanding, and insufficient use of prompts or “nudges” to remind users to engage [12]. In response to these challenges, we designed an mHealth solution (called “Tessellate”) dependent only on short message service (SMS) technology in order for workers in rural health centres (RHCs) to be able to report vaccine stock levels. In order to improve engagement, we incentivised users quasi-financially, by rewarding timely stock updates with free airtime, as well as prompting users to send stock updates by sending weekly reminder SMS messages. The objective of this study was to evaluate the potential of the Tessellate technology to offer a viable stock-tracking solution for RHCs in rural Zambia. This would be evaluated via a primary outcome measure of percentage-of-doses-tracked. The secondary objectives of the study were to establish if user engagement could be improved with increased airtime incentives or with increased frequency of SMS prompts.

## Methods

**Ethics statement:** this study was approved by the Zambian Ministry for Health.

**Study setting:** the study was conducted in the Kazungula District in the Southern Province. This area is served by 21 public RHCs which cover a population of over 105,000 people. The distance between the RHCs and the district pharmacy in Livingstone varied between 17-287km [13].

**Figure 1:**
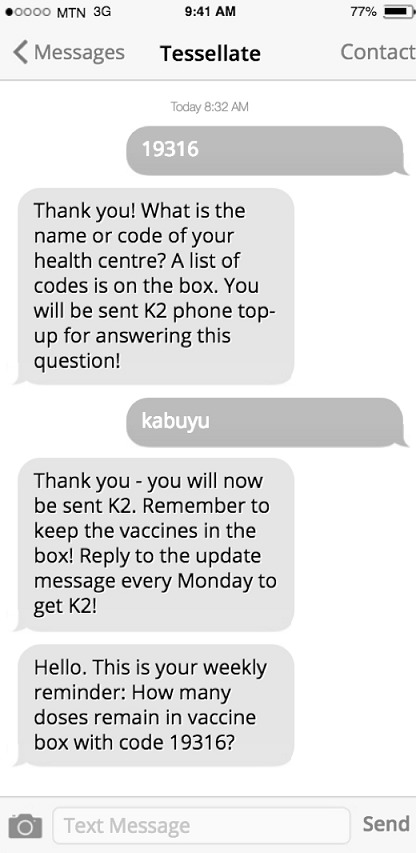
Example dialogue between community health worker (CHW) and automated short message service (SMS) replies

**Study procedure:** prior to commencement, the study team visited each of the 21 sites and conducted a 30-minute training session with the community health workers (CHWs) regarding the Tessellate system. They were given written materials and a poster to keep explaining the study, and a study telephone number which they could contact at any time for further information. For the duration of the study, between July and November 2017, identifying labels were placed on every box of pentavalent DTP vaccines at the district vaccine store in Livingstone. Each label displayed a unique 5-digit code. CHWs were asked to send this 5-digit code as an SMS to the project mobile phone number (printed on the vaccine box and on the written materials given during the site visit) when they collected the vaccines from the vaccine store; this “pick-up” message was the only unprompted message required. An automated reply was sent back, asking to which RHC the vaccines were being taken. Upon reply to this, an automated message was sent with a code for airtime. All users who had communicated a “pick-up” message were sent an automated message once a week asking how many doses remained in the box they had collected. If a user replied to this reminder message, they would receive a further airtime code. See Figure 1 for an example of an interaction between a user and the automated system. All messages received by the project mobile phone were automatically time-stamped and aggregated by a custom-written web platform which displayed recorded levels of vaccine stocks across all 21 RHCs in the district.

The primary outcome measure (percentage-of-doses-tracked) was defined as the total number of vaccine doses accounted for by the online platform at the end of the pilot period divided by the doses of vaccine which were collected from the district pharmacy during the same period. If no stock update message had been received regarding a particular box of vaccines in the past two weeks, or if no pick-up message had been sent by the CHW upon collection of the box, all remaining doses within the box were considered untracked. In order to achieve the secondary objective, namely, to establish if the response rate from users was affected by airtime incentive and/or reminder frequency, a 2 x 2 full factorial design was used, with airtime incentive (ZMK 4 vs ZMK 2, equivalent to US$0.40 and US$0.20 respectively) and reminder frequency (daily vs weekly) as factors. Importantly, the vaccine box labels did not display the airtime incentive amount or reminder frequency, and therefore these factors can have had no impact on whether or not CHWs sent an initial “pick-up” message. Therefore, for the secondary objective we measured median-time-to-reply rather than percentage-of-doses-tracked in order to measure any difference in messaging behaviour between groups. In boxes with “daily” reminder frequency, if the user did not respond to the weekly message asking for a stock update, then they were sent daily reminders until a reply was received. There were no such follow-up messages in the “weekly” reminder group. For this part of the statistical analysis, a 2-way analysis of variance (ANOVA) was used.

## Results

**Figure 2:**
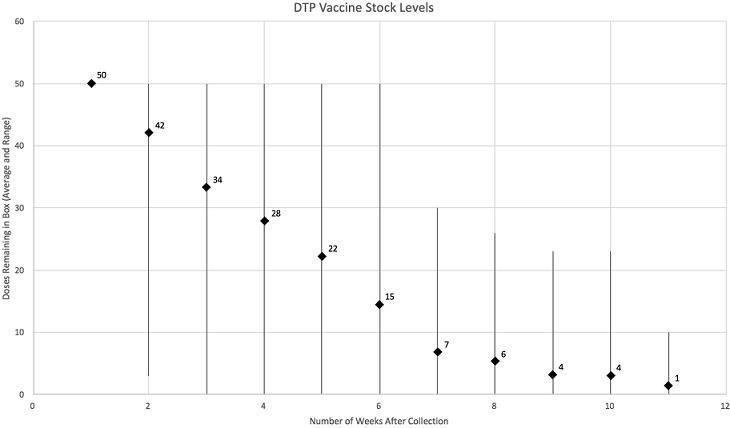
Variability of DTP usage rate

Of the 21 RHCs, responses were received from users from 10 facilities (48%); there were 13 unique users. Over the study period, a total of 7,900 doses of DTP vaccine were collected from the district pharmacy. Of these, complete tracking data were collected for 3,150 doses (39.9%). If an initial message “pick-up” message was sent to the platform upon collection of the vaccines, complete tracking data were collected for 93.8% of doses. Overall, there was no significant difference in average time-to-reply between the standard airtime incentive and double airtime incentive groups (13.3 hours (95% CI 2-24.5 hours) vs 7.5 hours (95% CI 3.6-11.5 hours) respectively, p = 0.42), nor was there a significant difference in average time-to-reply between the standard reminder and daily follow-up reminder group (16.4 hours (95% CI 4.3-28.5 hours) vs 4.0 hours (95% CI 1.9-6.1 hours) respectively, p = 0.08); although there was a trend for more timely replies in the daily follow-up reminder group, this did not reach statistical significance. There was no significant interaction between reminder frequency and airtime incentive on response time (F = 1.28(1, 68), p = 0.26). The mean airtime compensation sent per vaccine dose was ZMK 0.19 (USD$0.02) in the standard incentive group and ZMK 0.28 (USD$0.03) in the double incentive group. The collected data demonstrated that there was no consistent pattern with regard to the rate of DTP vaccine usage across facilities: the average time to use fifty doses (the number of doses in one box of DTP vaccine) was 10.5 weeks, however this demonstrated significant variability (Figure 2). Due to the low engagement rate, a post-hoc structured questionnaire was developed in order to identify the barriers to uptake from the 11 RHCs which did not send any update data to the project. All 11 RHCs were visited in order to administer the questionnaire and therefore the response rate was 100%. The majority (64%) of respondents cited inadequate understanding of the project as the main reason for their lack of engagement. On further questioning, it often transpired that key staff members had not been present on the training visits prior to commencement of the study period and therefore were not able to ensure that more junior staff bought into the project. A significant minority (27%) of respondents identified poor mobile phone signal as their reason for non-engagement.

## Discussion

The overall engagement rate found in this study - with just under half of RHCs choosing to participate - is broadly in keeping with existing evidence concerning the rate of engagement with stock tracking in Zambia [12]. Among those users who did choose to participate, however, the response rate was remarkably high, with most CHWs replying to stock update reminder messages in under a day. Within this active subgroup, near-real-time stock data were captured for 93.8% of doses, which is significantly above usual levels of reporting. It was unsurprising that there was a tendency towards more timely replies in the group who received daily reminder messages; this did not reach statistical significance in part because those in the weekly reminder group also replied promptly to their reminder messages. This study did not demonstrate any significant difference in engagement between standard and double remuneration groups. Previous studies in sub-Saharan Africa have shown that SMS-mediated financial incentives may increase patient adherence to medication [14], however there is ongoing and lively debate as to whether financial “bonuses” improve CHW performance in low-income settings [15, 16]. Research conducted in Zambia suggests that while conditional financial incentives may improve overall job satisfaction for CHWs, they do not significantly increase motivation [17]; this is consistent with findings in other sub-Saharan African countries [18, 19]. Finally, in similar settings it has been found that airtime is perceived to be less motivating than a cash transfer [20]; the findings from the present study would support the notion that non-monetary rewards do not significantly improve motivation in Zambian CHWs.

**Strengths and limitations:** one strength of this study was that it demonstrated the feasibility of using CHWs' own mobile telephones in order to record vaccine stock data. Previous pilot studies in which (smart) phones are distributed to CHWs are subject to the cost of phones as well as their loss, repair and theft - this significantly limits the scalability of such projects [21]. In the present study, the cost of incentivising users with airtime amounted to only USD$0.02 per dose. Moreover, as CHWs in sub-Saharan Africa often use their personal mobile phones for work-related communication [22], it seems sensible to take advantage of this existing resource. A further strength of the study was that its use of airtime rewards enabled a rapid positive feedback loop: allowing for lags in mobile communication, CHWs received their reward for stock updates typically within under ten seconds. Studies have shown that incentive schemes are frequently hampered by delays between positive behaviour and rewards [23, 24]; in the psychological literature, it is generally accepted that the more immediate a reward, the stronger the effect of positive reinforcement [25]. It is plausible, therefore, that the very high retention rate of users was in part due to the immediate receipt of the airtime reward.

One challenge for implementation included limited technological understanding of an automated SMS service - although clear instructions were given on the box, in written materials, and in the training session that users needed to send only the five-digit code to the project mobile phone number upon collection, a number of users sent lengthy natural-language messages explaining which vaccines they had collected and where they were being taken. It is possible that this limited understanding was a factor in the 52% of RHCs which did not send an initial “pick-up” message when they collected their vaccine stock. Multiple other studies conducted in Africa have also found that the feasibility of mHealth interventions in rural areas may be limited by technical barriers [26-29]. While a more comprehensive training course to explain the use of the SMS tracking system may mitigate this effect, it would also limit the scalability of the intervention. A second limitation of this study was that it did not capture the effect of vaccine tracking on rates and causes of vaccine wastage. This feature could be incorporated into the next iteration of the Tessellate technology: users could receive automated messages asking if any doses/vials from the boxes they collected have been wasted and, if so, for what reason. While this would undoubtedly gather useful information, there are a number of reasons why it may also be counterproductive. There is evidence to suggest that users of mHealth systems experience “message fatigue” [30, 31], asking users to report vaccine wastage as well as stock levels via SMS would significantly increase the number of messages required weekly. Moreover, due to the increased number of messages, the airtime costs of the project would more than double. Therefore, it may be more fruitful to send targeted messages to users who, for example, report erratic or disproportionately high vaccine usage rates.

## Conclusion

This pilot study found that in an active subgroup of CHWs, a novel, incentivised mHealth solution was able to collect tracking data for 93.8% of vaccines. However, due to the significant number of CHWs who did not choose to engage with the study, the overall accuracy of tracking data was low. The study demonstrates that an automated mHealth system can record vaccine inventory in rural areas at low cost without relying on paper records and using CHWs' own mobile telephones; however, more research is required to identify techniques to encourage reluctant users to engage with such a system - in the present study there was no evidence that compensating users with airtime sufficiently incentivises users.

### What is known about this topic

- According to the WHO, almost half of all vaccines are wasted;
- Improved logistics and stock management systems may generate improved efficiency of the vaccine supply chain;
- Few mHealth studies have been directed at vaccine supply chain management systems in Africa.

### What this study adds

- An incentivised mHealth system can track vaccine stocks in rural areas with 93.8% response rate from engaged users;
- Increased airtime incentives do not appear to increase response rates;
- More research is needed to identify methods to encourage health workers to engage in timely stock reporting practices.

## Competing interests

The authors declare no competing interests.
